# Convergence research for sustainable regional systems

**DOI:** 10.1016/j.isci.2025.113104

**Published:** 2025-07-22

**Authors:** Heejun Chang, Brian Roe, Murat Erkoc, Josiah Heyman, Katherine Foo, Debankur Sanyal, Debjyoti Banerjee, Richard Rushforth, Jaishri Srinivasan

**Affiliations:** 1 Department of Geography, Portland State University, Portland, OR, USA; 2 Department of Agricultural, Environmental and Development Economics, Ohio State University, Columbus, OH, USA; 3 Department of Industrial and Systems Engineering, University of Miami, Coral Gables, FL, USA; 4 Department of Sociology and Anthropology, University of Texas at El Paso, El Paso, TX, USA; 5 Department of Integrative and Global Studies, Worcester Polytechnic Institute, Worcester, MA, USA; 6 Department of Environmental Science, The University of Arizona, Tucson, AZ, USA; 7 Department of Mechanical Engineering, Texas A&M University, College Station, TX, USA; 8 School of Complex Adaptive Systems, Arizona State University, Tempe, AZ, USA; 9 Valley Institute of Sustainability (VISTA), University of California, Merced, Merced, CA, USA

**Keywords:** Environmental science, Environmental policy

## Abstract

Convergence research addresses societal and environmental challenges by co-creating knowledge across disciplinary boundaries, often in deep collaboration with extra-academic parties to ensure that the research process is solution-focused, actionable, and just. As part of National Science Foundation (NSF)-funded research projects on sustainable regional systems, we present multiple perspectives on conducting convergence research within the coupled social-ecological-technological systems framework. These projects explicitly engage extra-academic parties to co-design and co-produce knowledge throughout all stages of this participatory research. We introduce the 3 C’s model as a framework that links a research team’s Communication, Collaboration, and Creation decisions to the likely degree of convergence and societal change achieved. This model assesses the degree of integration among different social sciences, biophysical sciences, engineering, and other scientific disciplines, as well as extra-academic partners ranging from governmental agencies and industries to non-profit organizations and tribal communities. Integration in this context is assessed by its ability to situate local problems within broader systemic lenses, resolve conflicts among the priorities of partners, and conduct cutting-edge research that addresses real-world needs with actionable sustainability solutions. By synthesizing inclusive and just practices of conducting convergent research across a selection of research teams, we provide recommendations for academic researchers and administrators to encourage and advance convergence research in solving challenging sustainability issues.

## Graphical abstract

**Figure:**
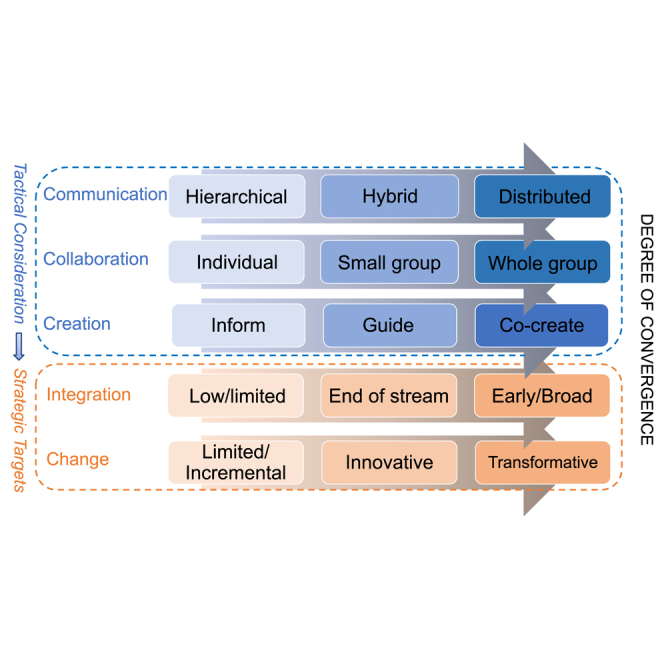

## Introduction

To tackle grand challenges to the sustainability of our society and environment, such as climate- and extreme weather-related events, food and energy security, and plastic recycling, convergence research is urgently needed.^1^ Convergence research is an approach to co-designing and co-producing knowledge and actions in addressing these complex and wicked social, environmental, and technological challenges by engaging with diverse parties, often transcending traditional boundaries of academic disciplines and organizations.^2^ Convergence research is needed to address a specific and compelling problem that can be defined within or outside the academic community.^3^ Additionally, convergence research “… shows deep integration across disciplines,” by purposely bringing diverse scholarly fields together to mingle disciplinary knowledge, theories, data, and methods through effective communication and synthesis strategies that identify and develop solutions to grand challenging problems. Initiated by biomedical sciences,^4^^,^^5^ convergence research endeavors have grown to include cyber-physical systems,^6^ brain science,^7^ and the NSF convergence accelerator as a way to bridge scientists and entrepreneurs.^8^

The importance of convergence research has also been recognized globally. In Europe, Science Europe^9^ emphasized its value, although more recent European Commission initiatives have tended to prioritize international collaboration over deep disciplinary integration.^10^ Convergence approaches grounded in coupled socio-ecological systems frameworks^1^ have gained traction globally. Examples include climate resilience planning in Southern African cities through the FRACTAL project, which employed embedded researchers and learning labs to co-produce actionable strategies with municipal stakeholders^11^; water-energy-food nexus research in Switzerland^12^; and renewable energy transitions in the Brazilian Amazon through participatory research with remote communities.^13^ In the Andes, co-production with indigenous communities has been central to identifying climate–biodiversity adaptation strategies that integrate traditional knowledge with ecological science.^14^ Additional efforts include Arctic system studies that draw on indigenous knowledge^15^ and community-engaged rural revitalization initiatives in Hong Kong.^16^ These international cases illustrate how convergence research is being increasingly used to address complex resilience challenges through inclusive, transdisciplinary, and regionally grounded approaches. However, existing literature is largely limited to convergence among academics^17^ and discusses extra-academic participants in a very abstracted and idealistic way. Thus, there is a need to discuss successful examples from existing convergence processes that consider convergence among a complex network of non-academic and academic parties addressing grand challenges inherently embedded in the functioning of regional systems.^18^

According to NSF,^19^ “*sustainable regional systems are connected urban and rural systems that are transforming their structures and processes collaboratively with the goal of measurably and equitably advancing the well-being of people and the planet*.” It is a useful lens through which to address socio-environmental grand challenges because it frames overlapping and intertwined ecological, technological, and social systems. Because urban systems depend on rural areas and their interconnected regional systems, we need to understand the symbiosis across the urban-to-rural spectrum.^20^^,^^21^ With funding from the NSF since 2021, multiple research groups have addressed sustainable regional systems (SRS) from a social, ecological, and technological systems perspective. The NSF call significantly increased the scale and scope of research studying urban-rural interfaces within sustainable regional systems. Meanwhile, the fundamental principles, processes, and practices of conducting convergence research in SRS projects vary substantially from one project to the other. Based on discussion at the NSF-funded Sustainable Regional System awardees conference in Alexandria, VA, in June 2023, this perspective article seeks to fill this gap by sharing experiences of six different projects to advance SRS science in convergence research (see Table S1). The NSF theme of sustainability in regional systems encouraged us to learn from and collaborate with people with practical knowledge of regional issues, interactions, and goals. The convergence of grounded knowledge and scholarly analysis provided creative synergies that enriched our learning processes. Effective dialogue, collaboration, and partnership beyond academia constituted our distinctive contribution.

Our goal in this article, then, is to share lessons learned about academic and extra-academic convergence–how this particularly challenging, but important kind of convergence was accomplished and how it could have been done better. We do not analyze specific data drawn from the projects since most of the projects were planning grants, with no programmed resources to collect original data to study convergence or to allow formal evaluation or comparison. We use projects as case studies to provide insight into what worked and what could have been better. Across the article, we provide the reader key frameworks for setting up convergence projects, and then experiences drawn from actually enacting those plans. To structure our discussion, we introduce the 3C model—Communication, Collaboration, and Creation—as a framework for understanding how research teams’ decisions shape the degree of convergence and societal impact achieved. From this, we offer initial conclusions about effective organization and practices of convergence, especially when bridging academic and extra-academic worlds.

The article focuses on three major elements of convergence research, drawing examples from several SRS network projects. First, we discuss the fundamental principles of convergence research by defining convergence approaches in comparison to traditional interdisciplinary and transdisciplinary research, and then propose a conceptual framework that explains potential factors affecting the degree of convergence research. Second, we discuss the process and practices of conducting convergence research, including cultural understanding, reflective processes, and norm changes. Third, we discuss case studies illustrating the complexities and lessons learned in extra-academic partner engagement and associated organizational behavior changes. Finally, the article summarizes the key findings and suggests recommendations for university researchers and administrators to advance convergence research and educational efforts needed to support convergence research.

### Principles of convergence research

The NSF, as part of its SRS request for proposals, challenged applicants to wield a convergence research approach to develop solutions to the multifaceted sustainability challenges such as water, food, and energy security and sustainability across regions and scales, confronting urban-rural systems across various geographies.^19^ Many past efforts in this space exhibited a limited scope, often targeting singular issues or risks. Given the highly variable spatial and temporal dynamics inherent in such challenges, there is a pressing need for comprehensive, collaborative, and robust planning that spans regions and aligns diverse interests.

To set the stage for defining and assessing the convergence research approaches wielded by our teams in support of creating more sustainable regional systems, we briefly review several modes of research that feature more than one discipline. Multidisciplinarity refers to research that includes the presence of multiple academic disciplines, though contributors largely stay within the confines of traditional disciplinary boundaries^22^; interdisciplinarity refers to the interaction of multiple disciplines with contributors seeking to coordinate linkages and harmonize outputs across disciplines^23^; and transdisciplinarity refers to the problem-driven integration of knowledge across disciplines that produces new, holistic knowledge (scientific merit) that is socially impactful (social relevance). More specifically, transdisciplinarity has two key components: (1) the (partial) dissolution of disciplines in the production of new scientific knowledge, and (2) some degree of “mutual learning between science and society.”^24^ Thus, transdisciplinary research co-produces knowledge in collaboration with extra-academic partners such as practitioners.^25^^,^^26^

Convergence research is similar to transdisciplinary research in its emphasis on developing solutions to compelling societal challenges by overcoming traditional disciplinary boundaries.^25^^,^^27^ Furthermore, convergence research emphasizes the creation of novel scientific knowledge as a fundamental outcome of the research process in addition to the generation of solutions to grand challenges.^27^ While not precluding knowledge co-generation between academic and extra-academic groups, scholarly convergence research does not necessarily require such interactions.^3^ However, we argue that, in the context of addressing challenges to the sustainability of regional systems, it is difficult to imagine robust solutions emerging without deep and meaningful knowledge co-generation with non-academic partners.

**Figure 1:**
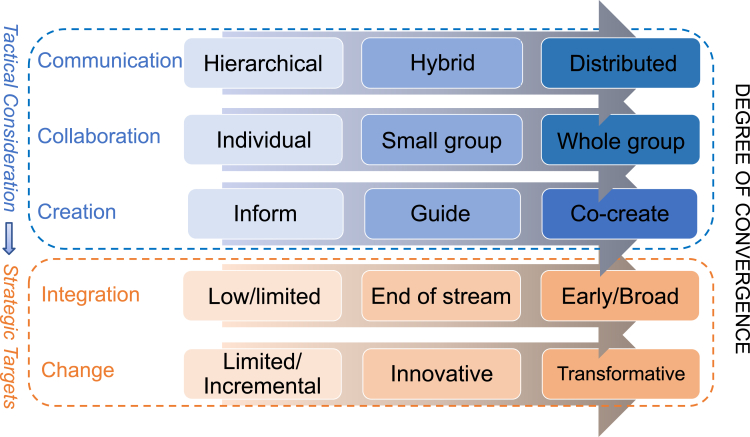
A conceptual framework showing potential factors affecting research teams to achieve the degree of convergence Notes: The elements inside the blue dashed line represent the 3 C’s of Communication, Collaboration, and Creation, which are tactical decisions teams make in pursuit of the strategic targets within the orange dashed line, which all feed into the degree of convergence achieved.

Given this critical engagement of extra-academic partners, we propose an effective convergence framework rooted in design thinking and systems science.^28^ This framework can serve as a catalyst for bringing together a diverse array of collaborators, including academic researchers, non-academic experts, policymakers, community leaders, industry representatives, and advocates necessary to address the critical societal problems challenging the sustainability of regional systems (Figure 1). We argue that following such a framework can increase the degree of convergence realized by SRS research teams, where we mirror Wilson’s^23^ vision of a continuum of progress along a “road to convergence.”

Figure 1 postulates communication, collaboration, and creation (3C) as tactical precursors that contribute to achieving the two key strategic elements that define convergence research: integration across disciplines and societal change. The framework is informed by design thinking and systems science,^28^ and conceptually rooted in organizational behavior, team science, and sustainability research. Communication affects trust and transparency in distributed teams,^29^ collaboration structures shape participatory knowledge production,^30^ and creation reflects co-production principles that involve extra-academic actors as full partners.^31^ While convergence teams may have a target for the desired degree of integration across disciplines and with non-academic partners, the chosen modes of communication, collaboration, and creation likely both shape the team’s ability to achieve these targets and also feed into revising these initially selected modes. For example, if hierarchical communication is initially employed but broader integration is desired, shifting to more transparent and distributed modes may be necessary. Similarly, collaboration involving all group members, including extra-academic partners as true co-creators, supports earlier and broader integration and increases the likelihood of achieving transformative changes.^32^^,^^33^ We hypothesize that higher levels of integration increase the probability of progressing from limited to incremental to transformative change.

Given this introduction to our proposed framework for understanding convergence research processes, we consider this framework in the context of sustainable regional systems efforts. At its core, convergence embodies a synthesis of ideas, methodologies, and solutions that transcend traditional boundaries by co-producing knowledge.^2^^,^^34^ In the context of addressing the intricate issues facing urban-rural systems, several key elements emerge as fundamental pillars of convergence: the 3 tactical C’s of communication, collaboration, and creation, and the strategic targets of integration and change. We now consider each lane of Figure 1 in the context of sustainable regional systems, starting with the tactical elements.

Effective **communication** serves as a cornerstone of convergence, fostering dialogue, understanding, and knowledge exchange among stakeholders.^32^^,^^35^ In the context of urban-rural systems, communication plays a vital role in articulating the complexities of these systems, conveying research findings to policymakers and communities, and soliciting and integrating input from diverse voices.^27^ By promoting transparency, accessibility, and inclusivity, communication enhances collaboration and ensures that research efforts are responsive to the needs and priorities of the communities they serve. Moreover, communication serves as a catalyst for building trust and forging meaningful partnerships, thereby laying the groundwork for sustained engagement and impact that contributes to sustainable regional systems.^32^

**Collaboration** emerges as a natural outgrowth of communication,^36^ enabling academic and non-academic partners to work together toward shared objectives in achieving sustainable regional systems. The convergence framework outlined above emphasizes the importance of bringing together researchers, experts, policymakers, community leaders, and advocates to co-design research and co-produce solutions for urban-rural systems. Collaboration transcends traditional boundaries and hierarchies, fostering a spirit of collective ownership and responsibility. Through collaborative endeavors, non-academic partners can leverage their respective strengths, resources, and networks to develop innovative approaches, mobilize support, and enact meaningful change.

**Creation** in convergence research represents the transformative potential of convergence, empowering extra-academic partners to generate new knowledge, methodologies, and solutions that directly address pressing challenges, ensuring tangible outcomes for both academic and extra-academic partners.^37^ For researchers, creation encompasses the development of research methodologies, analytical frameworks, and decision support tools that transcend traditional disciplinary boundaries. For extra-academic partners, it translates to implementable solutions such as policy frameworks, community-driven initiatives, and applied technologies that have direct impacts to the community. In the context of urban-rural systems, creation is the process through which collaborative research efforts generate actionable insights and strategies that inform both academic scholarship and community-based decision-making. By fostering a culture of innovation, co-production, and iterative learning, convergence research empowers diverse stakeholders to experiment with new approaches, refine solutions based on feedback, and adapt strategies to evolving environmental, social, and economic conditions. This process fosters resilience and ensures that convergence research leads to both scientific advancements and practical, on-the-ground improvements in urban-rural sustainability.^27^

Moving from tactical to strategic considerations in Figure 1, we first consider the role of **integration**, which is the foundation for convergence. Integration enables the assimilation of diverse insights, data, and expertise into a cohesive framework. It is a fundamental goal that research teams must achieve to effectively tackle complex challenges.^38^ Without integration, convergence research remains fragmented, limiting its ability to generate holistic solutions. In the context of urban-rural systems, integration is particularly critical due to the highly variable nature of sustainability challenges confronting urban-rural systems. Addressing these challenges necessitates comprehensive planning that synthesizes knowledge from social, environmental, and technological domains across regions.^39^ This requires the integration of knowledge from various disciplines, to develop holistic solutions that address the interconnected aspects of social, environmental, and technological systems.^40^ Integration will require a mix of solutions that must be assembled and scaled effectively. Building a regional plan requires both ex ante planning and ex post adjustments and multiple iterations to develop effective spatial and temporal models and solutions. Integration enables researchers and practitioners to move beyond siloed approaches and adopt a more synergistic and inclusive mindset, wherein diverse perspectives are valued and leveraged to achieve common goals, as demonstrated in the studies of drug trafficking activities and landscape changes^41^ and arctic stressors and changes.^15^

**Change** serves as the ultimate outcome of convergence, reflecting the tangible impact of research efforts on urban-rural systems and communities. Convergence aims to shift from localized solutions to transformative, system-wide approaches that address the root causes of complex challenges. Achieving this level of change requires challenging existing paradigms, reshaping norms, and transforming or dismantling entrenched power structures at individual, organizational, and systemic levels. By catalyzing positive change, convergence enables urban-rural systems to become more resilient, adaptive, and equitable, even amid uncertainty, inequality, and disruption.^42^ Ultimately, this process enhances the long-term sustainability and well-being of communities, ensuring that positive impacts extend beyond immediate interventions to shape future generations.

### Processes and practices of convergence research

Effective convergence research involves a continual process of learning about and building on the complexity around communication, different worldviews, and divergent timelines found among participants.^28^^,^^34^ Figure 1 embodies this process, and the challenges lie in the actual implementation strategies. Here we offer experiences and formulate guidance for effective practice. We argue that, given the nature of regional systems, both academic and extra-academic collaborators will be essential and coequal participants in convergence efforts. After all, convergence implies diverse sources, and when sustainability of a regional system is the focus, knowledge from community partners is critical to the development of implementable solutions.^27^ From these sources, convergence will not result in uniform approaches but rather context-specific approaches suited to different domains.^43^^,^^44^ This complexity can be heuristically divided into cultural differences and divisions of social structure, prevalent in both academic and non-academic worlds.

Culture identifies differences in meaningful symbolic frameworks, while social structure names the cleavages in interaction found in fields of unequal power.^45^ Both are kinds of complexity that challenge our capacity for meaningful and productive convergence. Hence, the process of convergence involves becoming aware of this complexity and then communicating and collaborating within it. Convergence does not erase these distinctions but instead admits their presence and builds within them in manners that forge trust, collaboration, respect, and shared missions.^46^

Contemporary social, environmental, and technological structures have been forged across histories of oppression, displacement, and marginalization, resulting in sharp political, economic, social, and cultural divides.^47^^,^^48^^,^^49^ Complex societies thus contain important differences among their sectors and communities in their socio-ecological-technological analyses and visions for sustainability.^50^ For example, rural-urban divides were inherent in the framing of our research program, and many other cleavages could be adumbrated. Moreover, ideologies obscure the meaning of past and present social inequalities for environmental research, governance, and stewardship, so that without a sustained and honest dialogue, people can be speaking past each other, without grappling with hard issues. Convergence, then, involves a difficult but vital process of uncovering and addressing internal ideologies and biases that block communication and collaboration, as well as strengthening formal organizational relationships.

Convergence processes in particular need to deal honestly and effectively with the gaps, past harms, and social barriers wrought by settler colonialism, slavery, and oppression of Black, Indigenous, and People of Color (BIPOC) communities. Collaboration norms may well reflect unequal power dynamics between academic and other research institutions and community partners. Likewise, there are significant inequalities among organizations and populations in communities themselves. In such contexts, effective convergence requires thoughtful self-consciousness and active equalization (albeit never complete) of power inequalities.^51^ Past and present relationships between academic and community partners, communication agreements, and inherited and new organizational forms all can promote meaningful, sustainable forms of convergence.^52^

To overcome the aforementioned challenges and achieve the 3 C’s in convergence research, here we propose 3 L’s (Listen, Learn, and Lead) model^53^ to operationalize the tactical elements (the first 3 C’s) that are critical for deriving integration and change, therefore, the overall degree of convergence. This 3 L’s model seeks to build trust and ensure equity and transparency in convergence research. As shown in Table 1, university researchers and non-academicians can work together by breaking their own assumptions in their day-to-day norms and practices, which can potentially lead to mutually agreeable practices at every stage of the convergence research process.

**Table 1:** The proposed 3 L’s model for implementing transparent and trustworthy convergence research

Parties	Listen	Learn	Lead
Academic researchers	• Become aware of the concerns and needs of extra-academic partners • Keep open diverse dialogues • Engage in active listening by removing purely academic lens	• Adapt research methods, timelines, and outputs to align with practitioners’ needs. • Become familiar with policy, industry, and community decision-making frameworks to enhance applicability • Learn about co-production principles to ensure actionable research	• Apply transformative theories by integrating extra-academic worldviews into research • Build accountable structures to ensure meaningful and sustainable partnerships • Facilitate capacity-building initiatives for extra-academic partners to engage in research
Extra-academic partners (including governmental and NGOs, private industries)	• Be willing to actively listen to other extra-academic partners • Keep open diverse dialogues among parties • Recognize research timelines and constraints	• Develop basic understanding on the scientific methods and evidence-based approaches used in research • Recognize that different partners operate on different timelines and integrate those constraints • Become familiar with fundamental concepts in convergence research	• Drive organizational and operational change based on co-produced knowledge • Co-lead initiatives in convergence research by working with academic partners • Shape research agendas by defining practical needs and knowledge gaps.

For example, in the “For Us, By Us” Ecology project,^54^ which examines regenerative food systems through reparative land relations, we embraced the 3 L’s in building trust with BIPOC farmers and land stewards in a few ways. First, listening and learning were enabled by selecting the core research team to be a university-community partnership with the Black Farmer Fund and Northeast Farmers of Color Land Trust. Second, the project developed intentional spaces to listen and learn from BIPOC farmers and land stewards, including interviews, farm visits, and participant observation of BIPOC farming events. Third, the core team embraced research participants’ worldviews to characterize their cultural ecological knowledge and farming practices as the basis of our For Us, By Us Ecology model.

**The For Us, By Us Ecology** project demonstrates the importance and complexity of relationship building to convergence research. Many potential collaborators and research participants turned down requests for interviews or farm visits. One refused because they felt uncomfortable with the project. Before interviews, several asked to learn more about the research, including research deliverables. Potential participants also asked about whether academic researchers would be committed to the movement and the shared vision beyond the project period. It is the ethical responsibility of scholars to invest in relationships with their community partners to build understanding, authenticity, and trust between them. Convergence research that is limited to academic funding periods risks undermining the long-term cultivation of value-aligned and reciprocal relationships over time and undermining the degree of change possible.

### Case studies in convergence research for sustainable regional systems

We report three case studies that exemplify the challenges inherent in convergence and processes to address them. These three projects’ topics span from food waste and microplastic pollution to community adaptation to sea level rise (SLR). These topics all address challenging social, environmental, and technological problems that require convergent thinking and approach in creating sustainable regional systems, thus meeting a key element of NSF’s definition of convergence research. Additionally, all projects engaged researchers from multiple disciplines, many of the researchers had worked in multi-, inter- and *trans*-disciplinary settings, providing the potential for deep integration across disciplines necessary to fulfill the second defining aspect of convergence research.

The RECIPES (Resilient, Equitable, and Circular Innovations with Partnership and Education Synergies) network focuses on wasted food. Globally, about one-third of harvested food is never eaten, which creates significant resource, environmental, nutritional, social, and economic consequences that are often disproportionately borne by historically marginalized groups.^55^ From the inception of RECIPES, active engagement of diverse non-academic audiences was critical to “… framing challenging research questions at inception, and fostering the collaborations needed for successful inquiry.”^56^ This inclusion of multiple universities and disciplines posed a distinct challenge to project leaders. The leadership team had to overcome communications challenges that arose among diverse academics seeking to transcend disciplinary perspectives while simultaneously integrating similarly motivated non-academic partners with critical knowledge, diverse timelines, and distinct internal accountability structures into the RECIPES network. Frustration can mount among extra-academic partners if academic partners bog down in cross-disciplinary boundary integration, as many extra-academic partners have either bridged disciplinary chasms in their own spheres or may not fully appreciate disciplinary divisions among academic partners.

Hence, an early initiative of RECIPES was to shape the emergent culture of this network, which, if left unchecked, could unconsciously import norms and principles from the various disciplinary and institutional affiliations of members. This systematic approach engaged RECIPES members in articulating key norms and principles they believed would best serve the convergence research process and be most likely to result in solutions to reduce and redirect wasted food to serve society. This was accomplished via broad participation in online ideation and consolidation sessions that were then honed into more concise and cogent sets of guiding principles and community norms^57^ (Table 2).

**Table 2:** RECIPES network guiding principles and community norms

Guiding Principles	Nested Community Norms
CO-CREATION OF KNOWLEDGE	Make knowledge, data, tools and resources easy to use, understand and access Learn from anyone and anything
PUTTING PEOPLE FIRST	Value and respect people, their skills, talent and knowledge
EQUAL ACCESS AND PARTICIPATION	Check your power and privilege
OPENNESS AND TRANSPARENCY	Communicate clearly, early and often
ACTIONS ARE GREATER THAN STATEMENTS	Follow through your commitments
RESTORATIVE, NOT PUNITIVE	Own your impact and seek to repair harm Be direct, caring and empathetic
REGENERATIVE, NOT EXTRACTIVE	Take space and give space Be reciprocal, whenever possible share your resources

*Source:* Agarwalla et al.^57^

The process resulted in seven guiding principles that provide high-level direction for Network actions, decisions, and products. Community norms describe how guiding principles are put into action and serve as the equivalent of a Code of Conduct. These are envisioned as a dynamic corpus with RECIPES members specifically tasked to collect and elicit feedback on the norms and update this guiding document. Each principle and norm consciously works against socio-cultural cleavages and power inequalities. Network members view the development and continued revision of the guiding principles and community norms as a shared commitment and a convergence process that is challenging, as it must persist over time, overcome tensions and contradictions that arise, and result in mutually satisfactory outcomes and meaningful action.

The MACRO-SETS (Microplastics Across the Columbia River Basin to the Ocean - Social-Ecological-Technological Systems) team addressed microplastic pollution in the geosphere, stressing the importance of integrating natural science to social science approaches in tackling plastic pollution.^58^ Environmental microplastic contamination is one of the grand challenges in the Anthropocene^59^^,^^60^ because microplastics have been found everywhere, from within deep sea creatures to protected wilderness areas at high altitude,^61^ negatively affecting the ecosystem health and regional economy. While there is a substantial variation in spatial and temporal variations of microplastic pollution,^62^ identifying factors affecting such variations and developing mitigation strategies requires a convergence approach. The project team members, who had prior experience working with scholars and practitioners in different disciplines, were well aware of potential challenges in conducting convergence research. Thus, they created a transparent and collaborative process to overcome these challenges at the onset of the project.

Communication and collaboration strategies were open and transparent. First, the team met regularly and organically identified four subtopics of interest (K-12 education, moss sampling, stakeholder surveys, and microplastic policy), with a pair of PIs working together to advance each component of the project with the engagement of relevant parties. The entire project team met monthly in the initial year to converge disparate ideas since the team had diverse academic backgrounds and expertise. The vital communication results were shared with the entire team at project meetings or in a Google Folder. Workshop agendas (e.g., variables of concern, workshop format) were co-identified by consulting key non-academicians through Zoom interviews or surveys before the workshops. Thus, collaboration between university researchers and non-academicians naturally happened while relevant parties with different backgrounds tackled solving the challenging problem of microplastic pollution to create products together, whether they were associated with designing survey questions, selecting sampling locations, or exploring alternative intervention policy scenarios.^63^

The project team recruited a tribal representative and municipal regulators who engaged in dialogues at the same discussion table at a workshop, which was held at the Native American Student and Community Center, allowing active listening and learning via small group discussions. As the project progressed, the research network’s connectivity increased over space and time with a growing number of nodes and tight connections between nodes by embracing different types of non-academicians. We emphasized the role of key informants who played critical roles in expanding the network connectivity, which is well documented in engaged research.^64^ However, the project was somewhat challenging in recruiting industry representatives, given the potentially sensitive issues of these industry partners to potential regulation.^63^

The CARES-GCR project (Convergent Adaptation to Sea Level Rise for Resilient and Equitable Rural and Urban Systems in the Gulf of Mexico Coastal Regions) tackled the challenge of sea level rise in the region. This initiative sought to bridge disparate perspectives across disciplinary and institutional boundaries, facilitating regional adaptation to sea-level rise across both short- and long-term horizons. To achieve this goal, the project aimed to develop models, mechanisms, and collaborative platforms to enable both vertical and horizontal integration and coordination. Vertical integration focuses on strengthening connections among communities, scientists, and decision-makers, ensuring that local knowledge informs policy and scientific advancements. Horizontal integration aims to enhance collaboration across interdependent infrastructure systems and neighboring regions, promoting regional-scale resilience.^65^ This two-way integration shifts the focus from localized solutions to system-wide optimization. The project’s vertical and horizontal integration strategies facilitated connections between scientists, policymakers, and communities, fostering a coordinated regional resilience approach. Similar multi-level governance frameworks have proven effective in addressing cross-scale sustainability challenges.^66^

As part of the early planning process, the project team organized an in-person charrette held in conjunction with an HBCU regional climate change conference. This event brought together a diverse group to formulate research questions and outline key components of the project framework, including research thrusts, stakeholder engagement strategies, and educational initiatives. This event set the tone for collaborative planning, emphasizing the importance of converging perspectives. A follow-up virtual charrette allowed participants to refine research thrusts, strengthen stakeholder engagement strategies, and educational initiatives. Insights from this continued dialogue laid the groundwork for subsequent planning and actions. Throughout the project, communication was a central pillar, structured around the 3C model. More than forty meetings, supplemented by email and video conference exchanges, facilitated meaningful collaboration between individuals from academia, non-profits, regional planning bodies, local governments, and community outreach specialists. This extensive network enabled cross-sector collaboration, forming a strong foundation for addressing the complex challenges of SLR.

The project team employed both the 3C’s and 3L’s models, ensuring that the project partners listened to and learned from one another’s expertise while also exploring ways to co-create new approaches for integrating their expertise into the broader climate adaptation strategies. Eventually, a network topology emerged that not only linked project thrusts to team members but also provided a structured framework in which they collaborate with coordinated goals and measurable outcomes. By fostering coordinated stakeholder engagement, inclusive participation, and multi-dimensional communication, the project has laid the groundwork for synthesizing research into actionable solutions and building long-term capacity for SLR resilience and adaptation.^67^

While substantially different in terms of scope, duration, and topic, these three exemplary convergence projects share some common lessons in conducting convergence research for SRS. First, convergence is not a single scientific measure but a fuzzy concept.^40^ Multiple levels of integration in theories, data, and methods are possible because convergence research boundaries continuously evolve as the project team members learn from each other while its network expands over time.^34^ Second, multiple factors contribute to the potential success of convergence research, although specific metrics for success can be plural. As discussed in the team science literature,^43^ the team composition, team dynamics, expected norms for communication and collaboration, and the ways to resolve conflicts all contribute to the success of convergence research.^68^ Third, convergence research led to emergent experiences for participants in the convergence research process. Convergence research has opened more (often unexpected) opportunities for all parties participating in the process. As convergence networks became larger and tighter, excitement increased, generating more concrete collaborative ideas at different stages, while offering transformative research opportunities to graduate students and junior scholars.^69^ We thus view convergence research as an evolving, continuous process rather than a final product resulting from a particular set of objectives. Our lessons are in line with other convergence research projects that have stressed the importance of team science at every research life cycle, appreciation of context-specific placed-based knowledge, educating and training the next generation of scientists, and epistemological and methodological integration by creating common goals and dataset.^2^^,^^16^^,^^18^^,^^41^

### Conclusions and recommendations

Humans confront numerous important social-ecological-technological challenges that demand creativity and wisdom to identify solutions. The demands are societal and transcend internal scholarly collaboration. We discussed the concept of convergence collaboration among scholars to deepen collaboration between academics and extra-academic members. University-community partnerships produce valuable new kinds of knowledge and action. For the regional sustainability concerns that drove our projects, and similar initiatives, this includes not only people with similar educational backgrounds now located inside and outside universities, but a wider range of participants from many walks of society. That experience will prompt university-affiliated scholars to reflect upon their wider social setting, while they support and engage with community members in their own investigations of sustainable regional systems. While non-academic constituencies vary enormously in wealth, power, and social standing, the counterparts with whom we engage are characteristically disadvantaged in many intersecting ways (class, gender, race, immigration status, and so forth). We think this matters; we challenge scholars to bring the vast resources and intellectual skills of academia to serve and collaborate with people who historically have been excluded from the “ivory tower,” and yet who hold important knowledge drawn from learned experience with social-environmental-technological challenges. They are also communities that will do the hard, practical work and will benefit the most from solutions emerging from convergence. Likewise, academic constituencies are similarly diverse; these university-community partnerships may strengthen the capacity of university-affiliated scholars to achieve equitable and inclusive collaborations, and they may support underrepresented scholars in accomplishing collaborations with community members with whom they share many social and cultural identities.

That convergence process, summarized as a set of ideal stages and goals in Figure 1, with supporting approaches provided in Table 1, and then described across the case studies, takes commitment and time. All need to feel mutual respect and commitment to the ups and downs of the process. Over time, this process creates a crucial quality of mutual trust, especially notable in contexts of previous separation and distrust. None of this is simple or easy! But it can be done with conscientious dedication and persistence. Out of that convergence emerge products of co-creation. Such results are valuable, indeed vital, and justify the long path to genuine convergence. But this process requires that we sustain convergence projects over time, to accomplish complex goals and genuinely demonstrate mutual commitment with regular reflective activities.^25^ Future work should validate the framework proposed in the study, including developing evaluation metrics of the convergence process and products.^18^^,^^70^^,^^71^

Convergence sustainability, in turn, will require fundamental organizational changes in higher education and funding agencies. We propose that institutional administrators consider three important, related areas to support convergence research and education: 1) updating grant management for improved community outreach protocols; 2) changing tenure and promotion criteria to support convergence research; and 3) embedding inclusion and just curricula in graduate education and faculty hiring and training (e.g., Box 1).

Box 1 **Implementing a Convergence Inspired Learning Module on Diversity, Equity & Inclusion (DE&I) in “Ethics and Engineering” Course**

Since the 1990s, the engineering curriculum at Texas A&M University mandated a core course on ethics (ENGR 482: Ethics and Engineering) for graduating seniors. ENGR 482 has been taught in each semester annually for 800–1200 engineering students, including in Engineering Study Abroad programs, while enrolling both domestic and international students from the host university overseas. The course was team-taught in each section by an engineer and a philosopher. Starting from Fall 2019, a research team implemented a new learning module on DE&I topics in ENGR 482. Inspired by the concept of convergence,^72^ this learning module was implemented as a part of an NSF sponsored project, on “Cultivating the Culture of Ethics in STEM.”^73^ Using the concept of 3 C’s and 3 L’s, the sub-topics in this course module on DE&I was developed by a team of faculty members from geographically separated multiple campuses, including Law School, College of Liberal Arts (Philosophy, Women & Gender Studies, Psychology & Brain Sciences), and the College of Engineering. The focus of the DE&I course module was to broaden the cultural horizon for the students, in terms of ethical infrastructure in organizations (e.g., mechanisms for reporting sexual harassment), personal conduct of engineers as individual entities in organizations (e.g., as diversity champions, advocates, and so forth), and case studies (e.g., sexual harassment case and the consequent change in company leadership). The team of investigators on this NSF project implemented their tasks based on the 3 C’s model, i.e., extensive discussions based on intensive literature reviews focused on the nuances of the topics that were eventually covered in the learning modules that became the cornerstone of convergence research on sustainable regional systems. The change in student attitudes was monitored using survey instruments that were utilized for obtaining student comments, both before and after the DE&I module was implemented in each section in each semester.^74^ The ultimate objective of the course module was to raise the awareness of the students on the different nuances of sexual harassment in STEM disciplines and to educate the students on the different dimensions pertaining to DE&I issues in engineering practices as well as to enhance their sensitivities to the value of having a diverse workforce.^74^

Grant management at many institutions can be improved. Regional-scale convergence research requires strong, reliable, and long-term community leadership. This is demonstrated in our 3L model, which is fundamentally rooted in building relationships with non-academic partners. As noted previously, convergence research often orients toward collaboration with underrepresented communities, and it is essential to invest resources and effort over extended periods to build mutual trust with them. Ultimately, institutional grant management offices decide whether this is possible and/or the extent to which this is possible. However, despite variations across institutions, the prevailing administrative requirements for community participation are onerous. The complexity of management protocols significantly hinders convergence research at multiple levels and in a variety of ways. The end result is that in some instances, the bureaucracy delays payments, disbursing payments over several months or even years. In the case of the For Us, By Us Ecology project, delayed payments strained multiple community interactions and ruined two key relationships. For convergence research to be viable, institutions must heavily reform their grant management protocols, collaborate across institutions to develop best practices, and work to uphold the ethical standards of human subjects research. By changing current bureaucratic structure and practices, institutions can provide the necessary operational coordination for convergence projects while improving organizational efficiencies.^75^

Promotion and tenure expectations for early-career faculty engaging in convergence research will need reform. Convergence integrates multiple scholarly disciplines and requires substantial time and resource commitment to activities outside traditional scholarship^76^ (Chang et al. 2020). As current promotion and tenure criteria often prioritize research outputs such as publications in prestigious journals, they may restrict researchers to a predetermined scientific niche. These criteria may not fully capture the scholarly contributions from cross-disciplinary convergence research, nor those of societal transformation. Also, it takes time to conduct convergence research and realize its impact, which again needs suitable policies. Academic institutions thus must address the necessity of convergence research, irrespective of scientific discipline, and re-frame promotion and tenure processes, incorporating metrics that value transdisciplinary collaboration and broader societal relevance^77^^,^^78^^,^^79^

There is a pressing need to acknowledge the importance of policy change as a legitimate outcome of academic research. This acknowledgment requires a cultural shift toward valuing broader impact alongside intellectual outputs, fostering a more holistic understanding of academic success.^80^ Academic incentives, then, need to reward more products and outcomes than simply publishing research articles. Many institutions have started to incentivize translational research that can produce actionable policies and practices for societal renaissance.^81^ Likewise, rising academics will need to be comfortable with–indeed, embrace–diverse, often historically underserved communities, crucial partners in convergence research. This will require genuine integration of Diversity, Equity, Inclusion, and Justice curricula in the preparation of future researchers (see Box 1). Additionally, by conscientiously recruiting BIPOC scholars as a cohort, universities can open new collaborative opportunities with underserved communities.^82^ By recognizing contributions to policy development through engagement with non-academicians, the implementation of research-informed interventions should be easier but more effective. By incorporating these changes, universities can better fulfill their societal needs of serving communities, fostering innovation, addressing pressing challenges, and contributing to broader communities in which they are embedded.

## Declaration of interests

The authors declare no competing interests.
