# Supplementary material for: Convergence research for sustainable regional systems
Source: iScience. 2025 Jul 22;28(8):113104. doi: 10.1016/j.isci.2025.113104 (PMC12343861; doi:10.1016/j.isci.2025.113104)
Supplement: Document S1. Tables S1 [file mmc1.pdf]

iScience, Volume *28*

Supplemental information

Convergence research for sustainable regional systems

Heejun Chang, Brian Roe, Murat Erkoc, Josiah Heyman, Katherine Foo, Debankur Sanyal, Debjyoti Banerjee, Richard Rushforth, and Jaishri Srinivasan

Table S1. Main characteristics of sustainable regional system convergence research projects examined in the study.

Project topic	PI team disciplines*	Non-academic partners*	Region of interest
Microplastic cycle and pollution	Geography, hydrology, marine ecology, biogeochemistry, engineering hydrology, environmental social sciences, geographical and environmental education	USGS, PNNL, Oregon Department of Environment Quality, Washington Department of Ecology, City of Portland, City of Gresham, City of Boise, Lower Columbia River Estuaries, NW Indian Fisheries Commission	Columbia River basin
Headwater dependent communities; Food-Energy-Water-Systems	Hydrology, civil and environmental engineering, fire ecology, human geography, law, sociology, sustainability science, community engagement, environmental informatics, indigenous planning, education, water resources science, economics, biology, disasters research, extension, soil science, atmospheric science,	Colorado Water, City of Santa Fe Water Division, Community of Mora, NM, Washington Department of Ecology, USFS, BLM, USFWS, South Central Climate Adaptation Science Center, FEMA, USGS, PNNL, Navajo Nation, Coalition for the Poudre River Watershed, Indigenous Land & Data Stewards Lab, Native FEWS Alliance, The Nature Conservancy, Trout Unlimited, Ayres Associates, Wright Water Engineers	Intermountain West
Sea-Level Rise Adaptation	Systems engineering, civil and environmental engineering, ecology, ocean engineering, urban and regional planning, environmental science and policy, sociology, economics, geography	Resilient Cities Catalyst, The CLEO Institute, The Data Center of Southeast Louisiana, Matagorda Bay Foundation, Concordia LLC, Local governments (Southeast Florida Regional Climate Compact, Miami-Dade County Office of Resilience, City of Mobile AL, Texas General Land Office)	Gulf of Mexico Coastal Region (GCR)
Transforming Wasteful Regional Food Systems	Design, Education, Engineering, Economics, Public Health, Chemistry, Nutrition, Psychology, Computer Science, Anthropology, Food Science, Environmental Science, Geography, Mathematics	National Resources Defense Council, ReFED, No Boundaries Coalition of Central Baltimore, DC Central Kitchen, Vivotein, CH4 Biogas, R.L. Jeffres & Sons, Eden Gives, Monroe County, NY (Dept. Env. Services), Black in Engineering, Radix Ecological Sustainability Center, Owareco, Biomass Controls, New York State Pollution Prevention Institute, Capitol Roots, Sky Farms, Women Advancing Nutrition Dietetics & Agriculture,	Multiple

		Natural Upcycling, Wegmans, World Wildlife Fund	
Shared destinies: Hydro-social infrastructures for community involvement and sustainability in fragmented border regions	Anthropology, Agriculture, Border Studies, Civil and Environmental Engineering, Political Science	RATES, Inc., Edinburg TX<br>El Paso Community Foundation<br>University of California San Diego Community Station, Tijuana, BCN, Mexico	U.S.-Mexico Borderlands
Regenerative and reparative food systems and land sovereignty	Geography, Landscape Ecology, Learning Sciences, Biogeochemistry, Agriculture, Business	Northeast Farmers of Color Land Trust, Black Farmer Fund	Northeastern US

*The list is not exhaustive, and readers may find the complete list of partners in each project website.

BLM = Bureau of Land Management; FEMA = Federal Emergency Management; PNLL = Pacific Northwest National Laboratory; USFWS = United States Fish & Wildlife Service; USFS= United States Forest Service; USGS = United States Geological Survey;
